# Clinical Prediction Models for Hospital-Induced Delirium Using Structured and Unstructured Electronic Health Record Data: Protocol for a Development and Validation Study

**DOI:** 10.2196/48521

**Published:** 2023-11-09

**Authors:** Sarah E Ser, Kristen Shear, Urszula A Snigurska, Mattia Prosperi, Yonghui Wu, Tanja Magoc, Ragnhildur I Bjarnadottir, Robert J Lucero

**Affiliations:** 1 Department of Epidemiology College of Public Health and Health Professions and College of Medicine University of Florida Gainesville, FL United States; 2 Department of Family, Community, and Health Systems Science College of Nursing University of Florida Gainesville, FL United States; 3 Department of Health Outcomes and Biomedical Informatics College of Medicine University of Florida Gainesville, FL United States; 4 Integrated Data Repository Research Services University of Florida Gainesville, FL United States; 5 School of Nursing University of California Los Angeles Los Angeles, CA United States

**Keywords:** big data, machine learning, data science, hospital-acquired condition, hospital induced, hospital acquired, predict, predictive, prediction, model, models, natural language processing, risk factors, delirium, risk, unstructured, structured, free text, clinical text, text data

## Abstract

**Background:**

Hospital-induced delirium is one of the most common and costly iatrogenic conditions, and its incidence is predicted to increase as the population of the United States ages. An academic and clinical interdisciplinary systems approach is needed to reduce the frequency and impact of hospital-induced delirium.

**Objective:**

The long-term goal of our research is to enhance the safety of hospitalized older adults by reducing iatrogenic conditions through an effective learning health system. In this study, we will develop models for predicting hospital-induced delirium. In order to accomplish this objective, we will create a computable phenotype for our outcome (hospital-induced delirium), design an expert-based traditional logistic regression model, leverage machine learning techniques to generate a model using structured data, and use machine learning and natural language processing to produce an integrated model with components from both structured data and text data.

**Methods:**

This study will explore text-based data, such as nursing notes, to improve the predictive capability of prognostic models for hospital-induced delirium. By using supervised and unsupervised text mining in addition to structured data, we will examine multiple types of information in electronic health record data to predict medical-surgical patient risk of developing delirium. Development and validation will be compliant to the Transparent Reporting of a multivariable prediction model for Individual Prognosis Or Diagnosis (TRIPOD) statement.

**Results:**

Work on this project will take place through March 2024. For this study, we will use data from approximately 332,230 encounters that occurred between January 2012 to May 2021. Findings from this project will be disseminated at scientific conferences and in peer-reviewed journals.

**Conclusions:**

Success in this study will yield a durable, high-performing research-data infrastructure that will process, extract, and analyze clinical text data in near real time. This model has the potential to be integrated into the electronic health record and provide point-of-care decision support to prevent harm and improve quality of care.

**International Registered Report Identifier (IRRID):**

DERR1-10.2196/48521

## Introduction

### Background

Hospital-induced delirium, defined as any episode of acute decline or fluctuation in attention, awareness, or other cognitive function, affects up to 56% of hospitalized older adults [1-3]. The experience of delirium can result in higher likelihood of hospital mortality, increased length of hospital stays, greater risk of 1-year mortality after discharge, functional decline, and increased caregiver burden [2-4]. The costs of hospital-induced delirium have ranged from US $16,000 to over US $64,000 per patient hospitalization, resulting in an estimated US $152 billion in national direct annual expenditures [5].

Clinicians, aging experts, patient advocates, and federal policymakers agree that there is a need to enhance the safety of hospitalized older adults through identification and reduction of iatrogenic conditions (ICs), such as hospital-induced delirium [6-8]. Advances in computing technology and availability of electronic health record (EHR) data—both structured, such as billing codes, and unstructured, such as clinical notes—present opportunities to assist health care systems and providers in more accurately identifying ICs and at-risk older adults [6,9,10].

Automated identification of conditions from EHR or other health data is not straightforward, even when biomedical ontologies such as the World Health Organization’s International Classification of Diseases (ICD) or the Systemized Nomenclature of Medicine–Clinical Terms are used [11,12]. Previous studies have shown that ICD code data captures a low percentage of delirium cases [13-16]. The notion of “computable phenotype” has been introduced to provide standardized, automated algorithms that make use of both structured and unstructured health data to identify conditions [11,12]. Automated methods, such as text mining, have shown promise for medical record reviews and retrospective identification of ICs, including delirium [17-23].

Computable phenotypes are vital to identification of conditions that can be used as “dependent variables” or “targets” for prediction models to discern high-risk patients. The use of clinical notes, such as nurses’ notes, has been shown to improve both computable phenotyping and prediction modeling [24]. There has been discussion about the importance of appropriately defining the outcome when developing a prognostic model for delirium; training a model to identify patients with a diagnostic code, as opposed to all patients with a condition, can result in a biased model. Researchers have suggested that incorporating clinical notes would increase the accuracy of a computable phenotype for delirium and improve a prediction model [25-27].

Registered nurses’ (RNs) progress notes, a type of clinical note, often contain important contextual factors and information related to patient safety that are not found in structured data fields [28]. The ability to process and analyze text data can fill gaps in practice-based evidence, provide contextual information, and improve the accuracy of prediction models [22,29,30]. The development of a research-data infrastructure that supports the use of both structured data and text data is critical for a learning health system aimed at improving care and patient outcomes [20,22].

Despite the value in text data, few hospital-induced delirium prediction models have included nurse-generated data, such as assessments and progress notes [2]. Prior studies have also emphasized patient factors which are not mutable, such as age and gender, even though clinical interventions can only be implemented with regard to variables that can be changed. Moreover, existing prediction models tend to have a high risk of bias, and the predictive value and generalizability of these models have rarely been validated using an external data source [31,32].

### Objective

Our long-term objective is to enhance the safety of hospitalized older adults by reducing ICs through a reliable and efficient learning health system. This will involve optimization of text and structured electronic data in a way that increases the reuse of clinical and administrative data for aging research, risk identification, and ultimately development of point-of-care technologies; and identification of the best ways to process and analyze text data generated by RNs to inform prospective identification of ICs in real or near–real time.

In this study, we will develop prediction models for hospital-induced delirium. In order to accomplish our objective, we will need to create a computable phenotype for hospital-induced delirium to be used as our outcome measure, design an expert-informed traditional logistic regression model to be used as a baseline model, leverage machine learning techniques to generate a model using structured data, and use machine learning and natural language processing (NLP) to produce an integrated model with components from both structured data and text data.

Building on our previous work with hospital-acquired falls [33,34], our study constitutes the first research effort to generate automated risk models of hospital-induced delirium by integrating RN-generated text data with existing structured patient, clinical, and administrative data.

## Methods

### Study Design

The study will use data from 2 academic medical centers in North Florida. Leveraging data from 2 medical centers will allow us to use data from 1 center for training and data from the other center for testing. Specifically, 1 medical center has about 1095 beds and 54,757 admissions per year, and the other medical center has about 603 beds and 23,685 admissions per year. We will include older adult (≥65 years) admissions to a medical-surgical unit between January 2012 and May 2021. We will obtain the data from the Integrated Data Repository (IDR).

The IDR was created to serve as a common source of information for clinicians, executives, researchers, and educators. The IDR enables new research discoveries as well as patient care quality and safety improvements through a continuous flow of information between the clinical enterprise and research community. The IDR currently consists of a clinical data warehouse that aggregates data from various clinical and administrative information systems, including the Epic electronic medical record. The clinical data warehouse contains demographics, inpatient and outpatient clinical encounter data, diagnoses, procedures, lab results, medications, select nursing assessments, comorbidity measures, and select perioperative anesthesia information system data.

### IC Task Force

We will develop a nurse-led IC task force whose purpose is to provide relevant clinical and technical expert knowledge to translate the use of electronic clinical data. In addition to focusing on delirium, members of the IC task force will also be involved in evaluating the expanded research-data infrastructure.

### Outcome

We aim to develop a computable phenotype that has greater sensitivity to identify patients with hospital-induced delirium than ICD codes alone. Along with using ICD code and medication data, we will incorporate nursing assessment documentation from structured data fields as part of our phenotype. Examples of the different types of data we will use include the ICD-10 code F05, the medication haloperidol, and the nursing assessment “observed/expressed feelings: agitated.” Expert clinicians will validate the phenotype; after evaluating performance, we will iteratively refine the phenotype and use it as our outcome measure.

### Structured Data Modeling

We will develop a clinical model to predict hospital-induced delirium, as defined by the (validated) computable phenotype.

We will compile a list of structured data variables to be used as candidate predictors of hospital-induced delirium, on the basis of a systematic review of the literature regarding model development and validation studies [32].

Clinical experts from our IC task force will help to select which predictors to use in a logistic regression model. We will also use machine learning methods, including decision tree, bagging, random forest, and adaptive boosting, to develop prediction models of hospital-induced delirium [35-39]. We will compare the performance of the models from machine learning techniques with our expert-informed logistic regression model. We are going to evaluate both discriminative ability and calibration; measures will include area under the receiver operating characteristic curve, balanced accuracy, sensitivity, specificity, and Brier score. We are going to use 1 admission event (encounter) per patient in order to avoid corelated instances.

### Text Mining

Supervised and unsupervised text mining approaches will be used to identify concepts related to risk factors, signs, and symptoms of delirium. For risk factors, we will be reviewing and adapting NLP pipelines developed to extract concepts related to risk factors for hospital-acquired falls. To identify and extract signs and symptoms of delirium, we will start by creating a reference thesaurus of keywords and phrases (ie, n-grams and sequence of consecutive keywords) thought to indicate signs and symptoms of delirium based on domain expertise and review of the literature. New themes that emerge from the data will be added to the reference thesaurus, which will be used to develop a guide for the manual annotation process. In total, 2 practicing bedside nurses will be trained as annotators to review progress notes and label phrases related to signs and symptoms of delirium. The entire process will be overseen by text-mining experts and clinicians.

We will use clinical named entity recognition (NER) to identify text patterns and attributes related to the signs and symptoms of delirium. The NER approaches are based on conditional random fields (CRF), a statistical method of sequence modeling [40,41]. The NER identifies the boundary (start and end positions) and semantic type for each clinical concept (eg, whether the concept is a laboratory finding, a medical condition, or something else). To develop the NER system, we will recruit annotators to manually identify the signs and symptoms of delirium from clinical notes as the gold standard. We will divide the annotations into a training set and a test set. The training set will be used to train the NER model and the test set will be used for performance evaluation. The performance of the NER model will be evaluated based on measures of precision and recall, including the F-measure. The model will also become a component of a text-mining pipeline to automatically extract and analyze RNs’ progress notes.

We will also develop a semiautomatic (unsupervised) method to discover previously unknown terms related to delirium from RNs’ progress notes using topic modeling which can support the generation of new hypotheses of risk factors or early signs of hospital-induced delirium. A topic refers to a cluster of similar words; for example, “confused, agitated, disoriented” could emerge as a cluster with the label “patient mental status.” We will apply latent Dirichlet allocation (LDA), a state-of-the-art topic modeling method which represents text files or documents as a collection of topics and assumes that each word is contained within one of these topics [42]. This probability-based approach will allow us to determine the optimal number of topics based on metrics of model performance. We will train an LDA model using the full collection of RN progress notes from one of the medical centers. The team will review the clusters of words generated by the LDA model and assign a clinical topic label based on the most frequently occurring words (this will allow us to organize the results and facilitate interpretation). Additionally, the results will be the foundation of an LDA model in our delirium text-mining pipeline. We will then generate a composite model of text and structured predictors by leveraging the structured data prediction modeling portion of our study.

In case more than 1 model has similar performance, the most parsimonious will be chosen. In order to integrate text data and structured data into 1 model, we will translate text into vectors (ie, a numerical representation) using Word2Vec models [43]. Word2Vec is a collection of models developed and widely tested to process a large body of text to produce a vector space, with each unique text attribute being assigned a corresponding vector in that space [43]. This mathematical representation enables the inclusion of text patterns in statistical and machine learning predictive models.

Finally, we will prepare a single data set that contains the converted textual data, and significant empirical and clinical factors from the structured data modeling. We will assign a vector value to each of the unstructured text variables. This way, the structured data variables and the text attributes will all be represented in the same format and can be fitted into the same vector space for prediction model development. We will then apply logistic regression methods with a LASSO (least absolute shrinkage and selection operator) feature selector and machine learning approaches including naive Bayesian classification, decision trees, and support vector machines. Interpretability and clinical significance are critical to understand the impact of different attributes on the overall prediction and clinical translation and will be carefully considered in choosing the best fitting model [44]. The performance of the prediction models will be evaluated by calculating sensitivity, specificity, and area under the curve using 10-fold cross-validation. To minimize false negatives (ie, failing to identify a person that truly is at risk of developing hospital-induced delirium), we will prioritize sensitivity; we will also aim to maintain a specificity of at least 0.9.

Once an integrated model is developed, we will externally validate it using a sample of patients hospitalized from January 2012 to May 2021 at the medical center which was not used for training the model. All work will be completed and results will be reported in accordance with the rigorous reporting and validation standard, the Transparent Reporting of a multivariable prediction model for Individual Prognosis Or Diagnosis (TRIPOD) statement, and its 22 associated items [45].

### Pipeline Infrastructure

We will integrate the clinical text pipelines by adding and linking them into existing components of the IDR. To evaluate the text-mining pipelines, we will carry out a testing procedure with two phases: (1) “white-box” testing—evaluating code coverage or the internal structures of modules within the research-data infrastructure, and (2) “black-box” testing—evaluating the functionality and scalability of the research-data infrastructure. For “white-box” testing, we will examine whether branch, path, and statement programming in the expanded IDR infrastructure is functional and error free. For “black-box” testing, RN members of the IC task force (see section above) will be asked to complete a series of tasks that involve querying clinical text data in the IDR instance of the Informatics for Integrating Biology and the Bedside (i2b2) computerized platform. These tasks might include identifying patients at high risk for delirium or patients with signs or symptoms of delirium. Tasks could focus on pulling data from structured EHR data fields, structured data derived from the NLP, or both. To assess functionality, we will measure perceived ease of use, processing speed, and errors reported. To determine scalability, we will assess memory usage and processing time of all “black-box” testing to identify bottlenecks or problem areas that could increase system load at scale-up. We will use an iterative testing approach to refine the integration of the text-mining pipelines until processing speed, memory use, and error reporting have reached acceptable levels.

### Ethics Approval

This project was approved by the University of Florida Institutional Review Board on February 11, 2019 (IRB201900208).

## Results

Work on this project will take place through March 2024. For this study, we will use data from approximately 332,230 encounters of 170,868 patients, including 6,980,470 narrative notes; these encounters took place from January 2012 to May 2021. We will create and validate a computable phenotype for hospital-induced delirium, develop prediction models for hospital-induced delirium using structured data and unstructured text data, and integrate text pipelines into the IDR. In addition, we will identify important nursing structured EHR data, curate data, and bring these data elements into the IDR to make them available for other future research projects.

Research findings will be disseminated through scientific conferences and peer-reviewed journals. Dissemination of the results and future studies (informed by this project) may expand practice-based evidence of hospital-induced delirium, which will improve clinical decision–making by clinicians and improve patient care in hospitals.

## Discussion

In this study, we will use both structured and unstructured EHR data to develop computable phenotype and prognostic models for hospital-induced delirium. By leveraging nurse-generated data, including structured fields from flowsheets and text from notes, we will identify more robust and accurate predictor and outcome measures, resulting in a highly accurate prediction model with less risk of bias than those from prior studies [31,32].

Creating multiple models will allow us to assess which data and modeling techniques make an appreciable difference. There are challenges associated with using text data, and not all EHRs contain easily accessible clinical notes. In our study, by comparing our structured data model with our integrated text and structured data model, we will be able to determine whether the text data significantly helps to predict which medical-surgical patients are at high-risk of developing hospital-induced delirium. Since the model with both unstructured and structured data will include the structured data model, we can use nested model criteria to compare their performance on the test set.

In addition, we will be able to compare our expert-informed traditional logistic regression model with models developed using machine learning methods. Even though models developed using machine learning techniques may be more accurate, they are also more difficult to interpret [44]. We will attempt to discern whether gain in performance is appreciable and if it outweighs the loss in interpretability.

Our study has some limitations; for instance, EHR data often has issues with completeness, accuracy, and complexity [11]. We will regularly consult with members of our IC task force so that we can better understand where and how clinicians usually document relevant data items in the EHR.

Findings from this study should allow us to develop and test a research-data infrastructure to support ongoing research on hospital-induced delirium, determine the scalability of our research-data infrastructure to OneFlorida, a state-wide PCORNet, and create synergistic relationships to test and translate our work in other health systems. This study will be a model for health care organizations to increase safe, effective care for the millions of older adults hospitalized every day.

## Abbreviations

EHR: electronic health record
IC: iatrogenic condition
ICD: International Classification of Diseases
IDR: Integrated Data Repository
LASSO: least absolute shrinkage and selection operator
LDA: latent Dirichlet allocation
NER: named entity recognition
NLP: natural language processing
RN: registered nurse
TRIPOD: Transparent Reporting of a multivariable prediction model for Individual Prognosis Or Diagnosis
